# Recent advances in green processing technologies for valorisation of eggshell waste for sustainable construction materials

**DOI:** 10.1016/j.heliyon.2022.e09649

**Published:** 2022-06-08

**Authors:** Blasius Ngayakamo, Azikiwe Peter Onwualu

**Affiliations:** Department of Materials Science and Engineering, African University of Science and Technology, Abuja, Nigeria

**Keywords:** Eggshell, Agro waste, Valorisation, Biocomposite and sustainable construction materials

## Abstract

Eggshell waste is among of the most abundant agro-waste material discharged from food processing industries. Despite the exceptional properties and several applications, eggshell is castoff in huge quantity without any further use. This review paper focuses on appraising the potential uses of eggshell waste as a feedstock for production of sustainable construction materials. The emphasis is on the need to exploit extensively eggshell waste as a partial cement replacement material in clay and cement-based construction materials. The physical-chemical properties of eggshell powder which describe its unique characteristics are discussed. The exploitation of eggshell waste in various construction materials have resulted into an overall improvement in the physical-mechanical properties. The results from reviewed work show that, the incorporation of 5–30 % of eggshell powder has developed green sustainable construction materials with properties that are within the range for the established engineering standards. In the current paper, it was indicated that the valorisation of eggshell waste has a potential to replace cement material for production of cheap and sustainable construction materials with improved engineering properties. Based on circular economy, valorisation is regarded to be a cost-effective solution to provide eco-friendly industrial raw materials while ensuring a waste free environment in the future.

## Graphical abstract

**Figure:**
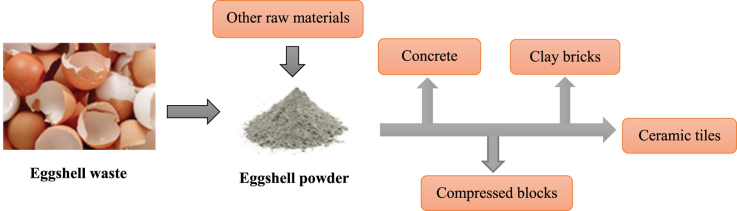

## 1 Introduction

In recent times, construction sector has been challenged with several factors particularly a sustainable exploitation of non-renewable natural resources that are finite and rapid infrastructure growth to produce construction materials [1, 2]. With the rapidly growing infrastructure development and an increase in urban population the demand of construction materials is substantially increasing [1, 3]. On the other hand, the production of cement-based construction materials has been spotted to have significant adverse impacts on the environment [2]. Currently, cement is one of the regularly used conventional construction material around the globe. However, the manufacture of cement demands utilization of non-renewable resources which generate carbon dioxide and other greenhouse gases (GHGs) that have caused major adverse environmental impacts [3]. Additionally, the production of cement materials requires significant amount of electrical and thermal energy to obtain the intended materials which corresponds to high building cost [1, 4]. It is reported that, in order to produce cement from non-renewable resources the process demand a calcination temperature of about 1450 °C which in turn discharges almost 0.85 ton of carbon dioxide for each 1 ton of cement manufactured [5]. The environmental concerns created by the production of conventional construction materials show that the construction industries are not sustainable.

Consequently, there is a need to develop sustainable construction materials that are cheap and eco-friendly with outstanding quality as cement in the long run. From the stand point of a sustainable green solution approach, several studies have reported the utilization of agro wastes such as sugarcane bagasse ash, seashells, rice husk ash, bamboo leaf ash, groundnut shells, cattle bone ash and fly ash as cement replacement [6, 7, 8, 9, 10, 11, 12, 13, 14]. On an environmental point of view, the recycling of agro bio-waste is regarded as the best eco-friendly approach to combat environmental problems associated to manufacture and utilization of cement which is currently mostly used in construction sector. The exploitation of bio-waste materials for replacement of cement is a practical solution to tackle environmental concerns caused by waste disposal in landfills. The conversion of bio-waste such as eggshell may minimize CO_2_ emissions since the calcination below 750 °C of a biomass does not generate CO_2_ which make the process to be economical, sustainable and eco-friendly [15].

Although there are indications that egg shell has the potential for replacing cement in construction materials, there is no study that brings all the advances in research together in order to guide future researchers and innovators on what works have been done. For that reason, this review work summarizes latest studies on the exploitation of eggshell as a cement replacement with a focus on the cutting-edge utilisation of eggshell powder in soil-bricks stabilization, clay bricks and green concrete. The work has been organized into six sections. The first section discusses challenges faced by construction sector in relation to high demand of construction materials and the potential impacts of conventional construction materials to the environment. The second section gives the fundamental concepts of agro-waste with a focus on the eggshell waste production, physical, chemical, and mechanical properties and potential uses in the third section. The fourth section presents work done on use of eggshell powder as a partial/full cement replacement in soil stabilization, clay bricks and green concrete as diverse sustainable construction materials. The fifth section discusses the challenges and way forward towards optimizing valorisation of eggshells bio-waste for construction materials. The sixth section gives conclusive remarks and research needs for effective valorisation of eggshells bio-waste in construction materials.

## 2 Fundamental concepts

Agricultural waste is a solid residue that is derived from crop farming and livestock production activities [16, 17]. Agricultural solid wastes comprise of crop residues, animal dung and silage effluents that are mostly recycled into energy to industrial sectors [16, 18]. The current trends show that, the production of agricultural waste including eggshells, rice husk and wheat straw to mention few is estimated to be 2 billion tons globally [19]. Furthermore, the expansion and massive production of agricultural produce in recent decades due to an increase in farming systems to feed the growing population indicates that more agro-bio-waste will be produced. Subsequently, several environmental strategies have been developed such as dumping agro wastes into landfills, composting and cremating agricultural residues have created adverse environmental impacts [20, 21].

Consequently, the disposal of agricultural residues has become an area of concern in most developing countries. The fact is that, when agricultural wastes are improperly handled they may lead to environmental hazards and pollution which may be harmful to public health by contaminating surface and underground water [22, 23]. This shows that, production of agricultural wastes from farming activities in large quantities may cause adverse environmental impacts due to unseemly disposal. Management of agricultural wastes is thus a compulsory global waste management strategy as accumulation of wastes may be of concern for animals, humans and vegetation [24, 25]. Hence, any type of agricultural waste should be properly managed and disposed of to protect environmental and public health [26].

The current emerging research works have demonstrated that valorisation of agro bio-waste is a viable and sustainable solution to tackle adverse environmental impacts caused by improper handling of agricultural wastes. The recycling of agro bio-wastes in construction sector may combat environmental pollution caused by conventional construction materials as well as environmental concerns of disposing agro wastes as described on environmental strategies. For instance, Sathiparan et al. [27], illustrated the potential use rice husk, coconut shells and peanut shells as the partial replacement in the production of cement blocks improved the strength and durability of the building material. As a result, several researchers have reported that, developing construction materials using agro wastes may result to more sustainable buildings. Basing on this prospect, the exploitation of agro waste for construction applications ensure environmental sustainability and minimize pollution and adverse environmental impacts in construction sector. The exploitation of eggshells is deemed to reduce agro bio-wastes which are disposed to the environment and thereby creating a sustainable environment free of wastes. In addition, the replacement of cement by eggshells powder will minimize CO_2_ emission from cement industries during the production of clinker.

### 2.1 Eggshell waste generation

Eggshell is an agro-waste material thrown away from food processing in homes and industries that has resulted to adverse environmental impacts in our surroundings [28]. The food processing industries comprises of large scale hatcheries and breaker plants which produce liquid, powdered and frozen eggshells for food producers, bakeries, schools, hospitals and fast food restaurants. As global population is surging up, similarly the production of eggshell wastes is estimated to increase that will demand high disposing costs for the large quantities of wastes released from food processing industries [29].

It is predicted that, 6.4 million tonnes of eggshells waste are castoff globally in landfills based on statistics released by the Food and Agriculture Organization (FAO) [30]. Eggshell as an agricultural waste may cause environmental pollution when it is not properly disposed of in the environment [31]. Although, eggshell waste has been utilized in soil remediation to rectify pH still large amount of it is discarded into landfill without any further use [32]. The unseemly disposal of eggshell wastes has become a concern due to development of urban pests and environmental odor [26, 31, 32]. Eggshell waste is highly generated from egg breaker factories than from households and restaurants that has resulted to substantial production of eggshells [29]. It is reported that, a modern egg breaker can process 188,000 eggs per hour whereby 30% of eggs are processed in liquids [33]. The global production of eggs exceeded 82.17 million metric tons in 2019 from 73.9 million metric tons in 2016. Since 1990 production of eggs worldwide in volume has improved over 100 percent [34].

It is projected that, by 2030 the production of eggs globally will be 90 million tons that will lead to a massive release of eggshells waste to the surroundings [35, 36]. The top 5 egg producing countries by the year 2020 are listed in Table 1 [37]. China is the largest egg producer with 37 percent of global production, followed by the USA with 7 percent and India with 6 percent. However, at continental level Asia is the largest producing continent with 64 percent of the global output [38]. In Africa, it is estimated that, 2, 367, 000 tonnes of eggs are produced annually which represent only 3.7% of global egg output. At country level, Nigeria is leading with 533,000 tonnes with Central African Republic, Comoros, Congo, Gambia, Guinea and Swaziland having less than 1000 tonnes per annum [39]. Consequently, high production of eggs corresponds to enormous generation of eggshell waste that are usually discarded and disposed of in various landfills. It is reported that, China is the leading eggshells producer with 24.8 billion kilograms in 2019 and it was expected to be more than 35 million metric tons by the year 2020 [40].

**Table 1:** Annual egg production by the top 5 countries in 2020.

Country	Number of eggs produced in (billions)
China	466
USA	109
India	95
Mexico	57
Brazil	54

### 2.2 Chemical composition and structure of an egg

Eggshell consists of 94% of calcite (CaCO_3_),1% of magnesium carbonate,1% of calcium phosphate and, lastly 4% of organic matter in the composition [41, 42, 43]. On the other hand, the colour of an eggshell does not indicate the quantity of calcium carbonate though 96–97% of calcium carbonate in brown eggshells with 4% of organic matter while 94% of calcium carbonate in white eggshell with 6% of organic matter [44, 45, 46, 47]. Eggshell however consists of hardened calcite and a shell membrane that is abundant in protein. The eggshell contribute to ∼12% of the total weight of the egg. An egg consist of 98% of the dry matter from which 93% contains ash matter and 5% crude protein and lastly 2% of water [40].

Structurally, an egg is composed of network of protein fibres that correspond to crystals of calcium and magnesium carbonate and calcium phosphate with other organic matter [40, 48]. Internally, an egg consist of calcified and shell membranes that involves inner and outer membranes that hold egg albumen and inhibit penetration of bacteria and moisture loss from an egg [49]. The skeleton of calcium carbonate has several structures with ∼10 nm particles which are arranged outward, the palisade and the mammillary layers with different which give the eggshell porous and rough structure [30, 50]. The normal fresh eggshell typically comprises of three layered structure namely the foamy cuticle layer which resembles a ceramic, the spongy middle layer and lamellar the inner layer [24]. On the other hand, calcium carbonate is regarded as the most essential constituent that is used for several industrial uses such as in gypsum and bone grafts applications [42, 43]. The calcite (CaCO_3_) is thus considered as a stable component that is extensively utilized as filler and a catalytic agent for different industrial usage [51].

### 2.3 Preparation and physical-chemical properties of calcined eggshell powder

The preparation of eggshell is generally preceded by washing or boiling in slightly acidic water, mechanical stripping and drying that can then be milled or calcined depending on the desired application. In preliminary stages, eggshell waste is washed and then boiled in slightly acidic water to remove impurities and the membrane attached to the shell [52, 53, 54]. The eggshell can then be air or oven dried before it is ground or calcined. The eggshell can be air or oven-dried for one to five days or 105–110 °C for 24 h depending on the intended use [53, 55, 56]. In the first approach, the dried eggshell can be calcined with the reported firing temperature in the range 600–1000 °C, during which a calcite (CaCO_3_) is decomposed to CaO and CO_2_ [57], [58] as shown in Eq. (1).

[1] CaCO_3(s)_ → CaO_(s)_ + CO_2(g)_

However, it is reported that at 900 °C, the entire eggshell is completely decomposed to CaO with a sharp weight loss due decomposition of calcite and other organic matters embedded in the eggshell [33, 59]. Table 2, shows comprehensive chemical composition of calcined eggshell powder from various studies. The concentration of CaO with an increasing temperature is reported to decrease due to formation of portlandite influenced by the interaction of CaO and moisture [27, 53] as shown in Eq. (2).

[2] CaO_(s)_ + H_2_O_(aq)_ → Ca(OH)_2__(s)_

**Table 2:** Chemical composition of calcined eggshell powder.

Composition in (wt.%)	Eggshell powder					
	[53]	[32]	[60]	[61]	[62]	[63]
CaO	79.28	50.70	52.10	52.10	46.69	64.83
Al_2_O_3_	0.34	0.03	0.06	0.03	0.12	0.13
SiO_2_	0.44	0.09	0.58	0.08	0.49	0.79
Fe_2_O_3_	0.004	0.02	0.02	0.02	0.32	0.06
Na_2_O	0.19	-	0.15	0.14	0.19	1.48
K_2_O	0.11	-	0.25	-	0.21	0.08
MgO	1.12	-	0.06	0.01	0.18	0.29
SO_3_	0.79	0.57	0.62	0.62	0.57	0.06
Cl	0.25		-		-	0.09
LOI	17.48	47.80	45.42	45.42	-	14.4

The calcined eggshell can be ground with steel drum or equipment in mill flow or by hand using mortar and pestle to fine powder that is sieved into micron size ready for use [52, 55, 64]. The literature has reported the use of sieve size of 75 μm and 90 μm [64, 65, 66, 67], though sieve size of 425 μm and 2.36 mm have been also used [53, 68]. The ESP is reported to have spherical shape at nano scale in the range 100–200 nm and irregular in shape in the range 20–200 μm [69]. The physical properties of ESP are summarized in Table 3 [69].

**Table 3:** Physical properties of powdered eggshell.

Physical properties	Eggshell powder
Shape	Spherical/irregular
Average particle size(μm)	1–155
Specific gravity	2.07–2.50
Bulk density(g/cm^3^)	2.50–2.62 [44], [56], [70]
BET surface area(m^2^/kg)	307–1400

## 3 Potential uses of eggshell

The industrial ecology method demonstrates the economic use of materials and energy in a sustainable way to minimize waste generation on the environment [71]. With emphasis on closed loop cycles (recycling and reuse) industrial ecology approach assumes waste as by-products and alternative feedstock for another industry [72, 73]. Consequently, solid wastes outside agricultural processing industries are transformed into the feedstock of another industry and used as secondary raw materials while minimizing utilization of virgin clay resources which are extremely used [71, 74]. The valorisation of eggshell waste can be a promising alternative not only to minimize environmental impacts but also for eco-commercial profit and a novel avenue for solid waste management.

**Figure 1:**
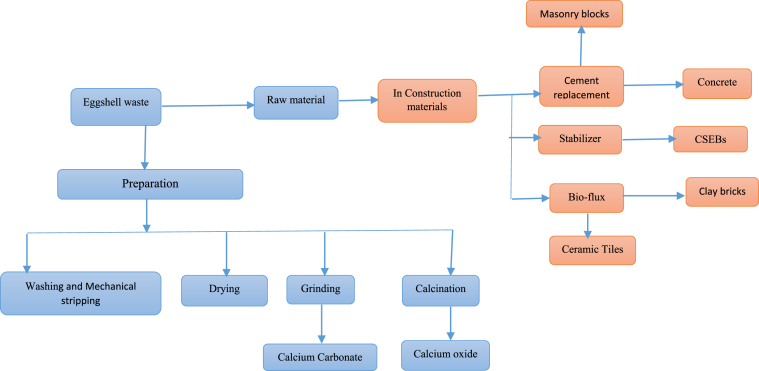
Preparation and potential uses of eggshell waste in development of construction materials.

Consequently, in recent times the valorisation of eggshells waste as an alternative raw material for production of value added-products is upsurging. For instance, eggshell waste has been used as an additive in calcium phosphate bio-ceramics [75, 76, 77], as calcium supplement [78, 79, 80, 81], adsorbent material [57, 82, 83], as a solid catalyst for biodiesel production [84, 85, 86, 87], bone graft substitute [28, 88, 89, 90], environmental remediation [91, 92], heavy metal removal [36, 93]. However, the sole application of eggshells in food and industrial rely main on the calcite component for making fertilizer, medicines and building materials. Despite the fact that, several sorptive and catalytic application of eggshell waste reported [94], currently there is an emerging interest on valorising eggshell in production of sustainable construction materials. Figure 1 summarizes the preparation and potential uses of eggshell waste for various applications in construction materials [3, 95].

## 4 Valorisation of eggshell in sustainable construction materials

Global valorisation of eggshells waste (ESW) has a promising prospect among technologists, scientists and engineers in recent times. The integration of eggshell waste has been regarded as the most cost-effective approach in minimizing the cost of construction materials [96]. Despite enhancing the physical-mechanical properties of several construction materials. The recycling and reusing of eggshells waste has proved to be both economic and environmental advantageous through effective exploitation of eggshells while reducing the cost of building materials for sustainable development. Therefore, by focusing on resolving the adverse environmental and health impacts caused by unseemly disposing of agro bio-waste, this review work focuses on utilization eggshells waste for developing sustainable construction materials namely; green concrete, clay bricks, ceramic tiles and stabilized masonry blocks.

### 4.1 Concrete

Concrete is the mostly used construction material in recent times that comprises of cement, fine and coarse aggregates and water derived from the natural resources [1, 54, 97, 98]. However, due to environmental concerns caused by cement during concrete production, researchers have reported the use of sugarcane bagasse ash (SCBA), groundnut shells ash as the alternative cement replacement materials. On the other hand, substantial studies have been done by applying eggshells powder in place of cementitious materials to minimize environmental impacts of the construction industry on climate change by reducing the release of GHGs due to cement production practices. Table 4, gives the summary of previous studies on eggshells powder replacement from 0-40 wt.% with a focus on compressive strength, splitting tensile strength and flexural strength.

**Table 4:** Mechanical performance of concrete with Eggshell Powder.

Reference	ESP variation(%)	ESP Optimum content	7 days			28 days		
			Compressive strength (MPa)	Split tensile strength (MPa)	Flexural strength (MPa)	Compressive Strength (MPa)	Split tensile strength (MPa)	Flexural strength (MPa)
[126]	5,10,15 and 20	15	27	2.4	-	38	3.2	-
[97]	5, 10, 15 and 20	15	-	-	-	48 · 36	-	10.1
[61]	5, 10 and 15	5	28	2.2	5.6		-	-
[104]	5,10 and 15	5	14.4	1.3	-	24	2.4	-
[127]	20,30 and 40	20	15.43	3.6	-	24.59	7.4	-
[128]	10, 20 and 30	10	-	-	-	78	-	8.6
[98]	5,10,15 and 20	15	34	-	2.8	37	-	3.2
[129]	5,10 and 15	5	18.5	2.2	3.2	35	3.3	4.8
[130]	0,5,10 and 15	5	15.1			20.19	2.23	3.51

From previous studies, the compressive strength, the splitting strength and flexural strength of the concrete are reported to improve with 5, 10, 15 and 15 % of ESP replacement. The mechanical properties of the concrete on 7^th^ and 28^th^ days under continuous supply of water (water curing) improved due to hydration and pozzolanic reactions which resulted to large amount of calcium silicate hydrate (C–S–H) which filled the voids and make the internal structure of the concrete to be consolidated thereby increasing the concrete strength [99, 100]. Consequently, it is obvious that the C–S–H gel is the essential strength enhancer and porosity reducer in concrete during cement hydration reactions [101]. As the amount of ESP increases result in extra amount of calcium hydroxide that contributes the conversion of liquid water into insoluble solid that increases slightly strength and water proofing ability of the concrete [102]. The prospective of water curing towards influencing the concrete strength has been nicely described by previous researchers [103].

On the other hand, as the ESP content increased, the mechanical properties of concrete (compressive strength, splitting tensile strength and flexural strength) decreased as reported by previous researchers [100, 104, 105]. This is due to the fact that, when calcium hydroxide is in excess results in deficiency in the amount of silica required to react with C–H to form the C–S–H gel which subsequently results in declining of concrete mechanical strength [2, 100].

### 4.2 Clay bricks

Clay bricks have been the common construction materials for a long time which by then allowed people to make huge buildings. This is due to their interesting physical-mechanical and thermal properties as well as durability [106]. However, the major drawbacks facing the extensive utilization of clay bricks are their limited mechanical strength, dimensional stability and high water absorption particularly when used in water logging areas. For that reason, there is a need to stabilize clay bricks to enhance their mechanical strength and reduce water absorption rate so as to make them stand up in competition with other modern conventional construction materials.

With the intention to save energy, stabilizing and improving the physical-mechanical properties of clay bricks fillers such as waste marbles [107, 108, 109, 110], lime [111] bone ash [112] have been utilized and reported from the published literature. On the flip side, several studies which report the integration of ESP in clay bricks have been summarized in Table 5. The addition of ESP has greatly improved the physical-mechanical properties of the clay bricks [53, 59, 113]. Despite the fact that, ESP has a low density, upon its addition to clay, the density of the clay was improved in both adobe, laterite and fired clay bricks. The improvement in physical-mechanical properties of clay bricks was attributed to the flux and filler effect of ESP which enhance vitrification and densification and the anorthite phase (CaAl_2_Si_2_O_8_) during a sintering process [53]. However, the sintering temperature of 1000 °C for a duration of 5 h has been described as the best firing parameters to enhance the physical-mechanical properties of the fired clay bricks [53, 113].

**Table 5:** Properties of clay bricks blended with Eggshell Powder.

Reference	brick	Other material	ESP variation(%)	Optimum ESP(%)	Water absorption(%)	density(g/cm^3^)	Hardness (HV)	Max. Compressive strength(MPa)
[59]	Fired	Granite and clay	5.10 and 15	10	12.2	1.76		3.12
[53]	Fired	-	5,10 and 15	15	11.1	2.1		4.8
[113]	Fired clay	-	5, 10, 15 and 20	20	14.36	1.75	8.79	8.28
[131]	Fired clay		10, 14 and 20	10	<25	-		14.38
[51]	Fired clay		5, 10, 15 and 20	20	14.36	1.75	8.79	8.28

### 4.3 Ceramic tiles

In recent times, ceramic tiles are the most widespread construction materials in developing countries [114, 115]. Due to technological advancements, ceramic tiles are currently regularly used construction materials in developing countries to improve the housing standard and infrastructural sector [116]. Consequently, ceramic tiles have several range of applications at the present time particularly in the contemporary societies and the demand is expected to increase in the future [117]. Thus, a substantial progress by the ceramic tiles industries in recent decades has been attained in terms of production capacity and manufacturing technology. The tremendous development of ceramic tiles industries, has resulted to huge intake and overexploitation of raw materials that have subsequently led to adverse environmental impacts due to enormous production of waste.

As a result, researchers have demonstrated the potential use of wastes as alternative raw materials to replace traditional raw material (feldspar as a flux) without adversely affecting the properties of ceramic tiles. On the basis of circular economy, the incorporation of Eggshell powder as a flux for the production of ceramic tiles was summarized in Table 6. With the addition of ESP from 0- 20% from the published works the physical-mechanical properties of the tiles gave the optimum values due to filler effect of the ESP content and the firing temperature [118, 119]. Freire et al. [119], reported that, the change in physical-mechanical properties was attributed to decomposition of calcite into CO_2_ outside the specimens. However, the calcite decomposition was revealed through Scanning Electron Microscopy microstructural characterization that which was dominated by high degree of porosity in the fractured surfaces [120].

**Table 6:** Properties of ceramic tiles with Eggshell Powder as a flux.

Reference	Tile	ESP variation (%)	Optimum ESP content(%)	Other materials (%)	Water absorption (%)	Bulk density (g/cm^3^)	Flexural strength (MPa)	Compressive strength (MPa)
[119]	Wall	15	15	Red clay and quartz	22.71	1.65	15.29	-
[132]	Roof	10, 15 and 20	15	Clay and Plastic	25	-	7.97	0.125
[133]	Floor	9	9	White cement and silica	3.1	2.43	4.5	17.95
[118]	Floor	10 and 20	20	Plastic and sand	0.15	1.83	11.7	-

### 4.4 Compressed stabilized blocks (CSBs)

Masonry construction was introduced ca. 5000-3500 BC, the time when clay bricks and alluvial deposits were used in construction applications [111, 121]. However, due to environmental impacts caused by fired clay bricks. The compressed stabilized blocks (CSBs) are deemed to overcome environmental issues associated with fired clay bricks production that involves CO_2_ emission which leads to acid rain and global warming [111]. Consequently, when the compressed stabilized earth blocks are utilized, the CO_2_ emission and adverse environmental effects can be controlled and minimized greatly. Although, the unfired clay bricks lack strength and durability hence mechanical and chemical stabilization are essential to reduce voids and compacting the green soil construction material. On this perspective, mechanical and chemical stabilization is commonly used to improve the mechanical strength of the CSEBs whereby lime and cement have been the most used stabilizers [122, 123]. Although, cement is the common earth stabilizer in recent times but the energy consumption and high CO_2_ emission discourage the use of cement [124]. Therefore, this review work focused on the potential utilization of Eggshell powder as cement replacement material in stabilizing and improving the mechanical strength of CSBs and other masonry blocks after 28 days of curing as shown in Table 7. The literature shows that, by incorporating eggshell powder into soil stabilized blocks resulted to an improved mechanical strength due to an increased consolidation influenced by the filler effect of ESP which filled the voids and decreased the open porosity [32, 125]. In addition, an increase in compressive strength is due to cation exchange influenced by pozzolanic reaction and flocculation-agglomeration after adding eggshell powder. During pozzolanic reaction silica, alumina and calcium hydroxide reacts to form calcium aluminate hydrate (C-A-H) and calcium silicate hydrate (C–S–H) respectively the cementitious materials which influence the mechanical strength of the CSBs [2, 123] as shown in Eqs. (3), (4), and (5).

[3] CaO_(s)_ + H_2_O_(aq)_ → Ca(OH)_2__(s)_

[4] CaO_(s)_ + SiO_2(aq)_ → CaSiO_3_.2H_2_O_(s)_

[5] Ca(OH)_2(aq)_ + Al_2_O_3(aq)_→ CaO·Al_2_O_3_.H_2_O_(s)_

**Table 7:** Properties of CSBs with eggshell powder.

Reference	Type	ESP variation(%)	ESP Optimum content	Other materials	28 days		
					Water absorption	Apparent density (g/cm^3^)	Max. Compressive strength(MPa)
[62]	Sandcrete	5, 10,15 20,25,30,35 and 40	30	Sand and cement	-	-	4.7
[32]	Soil-cement block	10, 20 and 30	10	Soil and cement	9.0	2.00	4.8
[125]	Soil-cement block	10, 20 and 30	10	Soil, cement and welding flux sludge	9.5	1.85	4.85
[63]	Laterite-block	10,20, 30 and 40	30	Laterite	3.94	2.04	2.87
[134]	Laterite-block	2,4,8 and 16	2 and 4	Sawdust ash and laterite	-	1.75	1.2

In the above reaction, calcium oxide (CaO) from the ESP reacts with water to form calcium hydroxide known as portlandite (Ca(OH)_2_) which reacts with silica (SiO_2_) form calcium silicate hydrate (CaSiO_3_·2H_2_O) or calcium aluminate hydrate (C-A-H) which have good cementing properties responsible for the strength [59].

## 5 Challenges in valorisation of eggshell waste

Eggshell waste has become the most copious agro-waste material discharged from food processing industries. Regardless of its unique properties and several applications eggshell is discarded in huge amount without additional usage. Consequently, eggshell waste has become a 15^th^ ranked agro-waste material that is regarded to have caused adverse environmental impacts as a result of its decay when disposed of in landfills [30]. Despite, the substantial studies on valorisation of eggshell waste in developing sustainable construction materials, a sector is still faced by myriad of challenges due to:

• Lack of sophisticated technology to harness eggshell waste. The absence of suitable processing technology tends to hinder the valorisation of the eggshell waste in developing countries. The outdated processing equipment and lack of essential spare parts has greatly affected the processing capacity of the eggshell raw material.
• Shortage of well-trained human resource in biomass valorization. The deficiency of well-trained scientists and researchers has adversely affected extensive sourcing and exploitation of the available eggshell waste for sustainable development of construction materials.
• Insufficient funding in R&D capacity building. In most developing countries sourcing of funds for R&D capacity building is very difficult. For that reason, R&D capacity building in our research institutes remains weak and subsequently the development of products from the available raw materials is adversely affected.
• Unavailability of raw materials on sustainable basis. The demand and a sustainable supply of agro raw materials for large scale production is a matter of concern in the developing countries. Consequently, due to shortage of raw materials supply has significantly contributed to the closure of most processing industries.
• Outdated policies in developing countries that fail to comply with new laws and regulations particularly in raw materials exploration and exploitation hence causing uncertainties in production and market prices. As a result, due to inconsistency in policies leave industrial organizations and local manufacturers at risk that subsequently affect the valorisation of agro raw materials.
• Weak linkages between research and industries. Research-industry linkage is aimed at enabling industries to have access of quality research produced from universities or research institutes to foster new inventions to enhance economic growth of industries and societies at a long run. However, in most developing countries there is a weak research-industry link and both organization appear to be operating independently. Therefore, there is a need to bridge the gap to foster research-industry ecosystem.

## 6 Conclusion

This article has offered an overview of the potential uses of eggshell waste for production of sustainable construction materials. The valorisation approach and integration of ESP in the production of concrete, clay bricks, ceramic tiles and compressed stabilised blocks (CSBs) has improved their engineering properties thereby ensuring their sustainability, minimizing costs and enhancing environmental conservation. The partial replacement of ESP from 5-30 % resulted in an improved physical-mechanical properties of cement based materials (concrete and sandcrete) and clay-based materials (clay bricks, CSEBs and tiles). However, excessive addition of ESP led to the declining of mechanical strength of the selected materials. The current paper shows that; valorisation of ESP is a promising approach for the partial cement replacement in the manufacture of sustainable construction materials. Even though, eggshell powder has unique chemical properties with high content of calcium oxide/calcite that make it as a potential cement replacement material for production of sustainable construction materials. However, there are some areas which need to be addressed.

• Several studies have been done on the potential application of ESP in concrete, clay bricks, CSBs and less in ceramic tiles. Nevertheless, further studies should be carried out on durability of ESP blended construction materials due to vulnerability of calcium compound derived from ESP to corrosive environments.
• Advanced study is required to investigate the potential causes and prevention of lime blowing in ESP blended construction materials to avoid their unnecessary deterioration upon exposure to air.
• In a sustainable solution approach, to save energy and reduce GHGs emission the optimum calcining temperature for eggshell should be recommended to avoid the adverse environmental and health impacts that may occur as a result of calcining eggshell at high temperature.

Therefore, valorisation of eggshell waste as a partial cement replacement towards green technologies will reduce the emission of GHGs during cement production. The valorisation approach can curb and resolve the problems associated with the disposal of agro bio-waste and their adverse environmental and health impacts to our surroundings.

## Declarations

### Author contribution statement

All authors listed have significantly contributed to the development and the writing of this article.

### Funding statement

This research did not receive any specific grant from funding agencies in the public, commercial, or not-for-profit sectors.

### Data availability statement

Data will be made available on request.

### Declaration of interests statement

The authors declare no conflict of interest.

### Additional information

No additional information is available for this paper.
